# The Use of Radiation Therapy in the Management of Selected Patients with Atypical Lipomas

**DOI:** 10.1155/2013/485483

**Published:** 2013-01-15

**Authors:** Josephine Kang, Maikel Botros, Saveli Goldberg, Christine Giraud, G. Petur Nielsen, Yen-Lin Chen, Kevin Raskin, Joseph Schwab, Sam S. Yoon, Francis J. Hornicek, Thomas F. DeLaney

**Affiliations:** ^1^Harvard Radiation Oncology Program, Boston, MA 02215, USA; ^2^Flushing Radiation Oncology Services, Queens, NY 11354, USA; ^3^Department of Radiation Oncology, Medical College of Wisconsin, Milwaukee, WI 53226, USA; ^4^Department of Radiation Oncology, Massachusetts General Hospital, Boston, MA 02114, USA; ^5^Center for Sarcoma and Connective Tissue Oncology, Massachusetts General Hospital, Boston, MA 02114, USA; ^6^Department of Pathology, Massachusetts General Hospital, Boston, MA 02114, USA; ^7^Department of Orthopaedic Oncology, Massachusetts General Hospital, Boston, MA 02114, USA; ^8^Division of Surgical Oncology, Department of Surgery, Massachusetts General Hospital, Boston, MA 02114, USA

## Abstract

*Background and Objectives*. Atypical lipomas are uncommon, slow-growing benign tumors. While surgery has been the primary treatment modality, we have managed some patients with radiation (RT) as a component of the treatment and have reported their outcomes in this study. *Methods*. A retrospective review of all cases of extremity and trunk atypical lipomas in The Sarcoma Database at the study institution was conducted. *Results*. Thirteen patients were identified. All patients underwent surgical resection at initial presentation and received pre- or postoperative radiation for subtotal resection (*n* = 2), local recurrence (*n* = 8), or progressive disease (*n* = 3). The median total radiation dose was 50 Gy. Median followup was 65.1 months. All patients treated with RT remained free of disease at the last followup. No grade 3 or higher late toxicity from radiation was observed. No cases of tumor dedifferentiation occurred. *Conclusion*. For recurrent or residual atypical lipomas, a combination of reexcision and RT can provide long-term local control with acceptable morbidity. For recurrent tumors, pre-op RT of 50 Gy appears to be an effective and well-tolerated management approach.

## 1. Introduction

**Figure 1:**
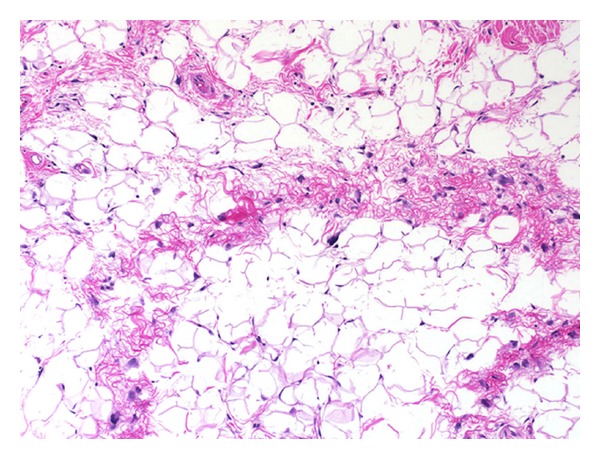
Histologic presentation of atypical lipoma. Atypical lipoma composed of mature fat and fibrous tissue with scattered, enlarged, hyperchromatic cells.

The term atypical lipoma was first introduced in 1974 [1] to describe lobulated, well-circumscribed, large lesions that are histologically characterized by mature adipocytes of varying size and scattered atypical stromal cells with hyperchromatic nuclei (Figure 1) [2, 3]. Atypical lipomas are slow-growing tumors that typically affect the extremities and trunk. In the absence of a dedifferentiated component, atypical lipomas do not metastasize and pattern of failure is predominantly local [3–11]. The primary treatment modality has been surgical resection, with reexcision for recurrent lesions.

The role and utility of preoperative or adjuvant radiation in management of these lesions is not well known. We have managed some patients with recurrent or subtotally resected tumors with radiation as a component of the treatment. In this study, we review the outcomes of thirteen patients with atypical lipoma treated with radiation therapy (RT) at the Massachusetts General Hospital during the years of 1995–2010.

## 2. Methods

A retrospective review of all cases of atypical lipoma treated with radiation at the Massachusetts General Hospital was conducted. Patients were excluded if RT was completed after January 2010. Thirteen consecutively treated patients were identified, initially diagnosed between 1987 and 2007. Median age at diagnosis was 54 (range: 36–76 years). 

With the exception of one chest wall lesion, all lesions were located in the upper (*n* = 4) or lower (*n* = 8) extremity. Of the lower extremity lesions, seven were in the thigh and one was in the calf. Of the upper extremity lesions, two were in the forearm, one in the deltoid, and one in the proximal arm. 

Details of each patient and treatment are shown in Table 1. Tumors were diagnosed based on reported histologic features after pathology review at Massachusetts General Hospital. Toxicity was graded based on Common Terminology Criteria for Adverse Events v4.0 (CTCAE). 

Gross total resection (GTR) was defined as tumor resected with negative margins. Subtotal resection (STR) was defined as gross residual disease. Local recurrence was defined as regrowth of tumor after a gross total resection. Progressive disease was defined as growth of residual tumor after STR.

All the patients underwent surgical resection at initial presentation, either GTR (*n* = 6), STR (*n* = 4), or extent of resection unknown (*n* = 3).

Of patients with GTR, local recurrence was identified at a mean of 58.5 months (range, 20.0–133.8 months) following resection. The locally recurrent tumor was treated with either preoperative RT (50 Gy in 2 Gy fractions) and GTR (*n* = 3), or resection followed by postoperative RT (50–66 Gy in 2 Gy fractions) (*n* = 3). 

Of the group of four patients with STR, two were monitored clinically and treated after evidence of disease progression, and the remaining two were treated adjuvantly with radiation for the following reasons. The first adjuvantly treated patient had atypical lipoma of the chest wall, and underwent tumor excision at an outside institution with gross residual disease. He presented with limb paresthesias prior to resection. He was recommended radiation followed by reexcision over observation due to his symptoms, and also because it was felt that a local recurrence would be difficult to resect given the tumor location. He received 50 Gy in 2 Gy fractions, followed by gross total excision of residual tumor. A second adjuvantly treated patient had subtotal excision of tumor that was overlying the sciatic nerve. Adjuvant radiation to 60 Gy in 30 fractions was administered due to concern of residual disease left within the region of the sciatic nerve. The remaining two patients, who were monitored clinically after STR, ultimately required RT with (*n* = 1) or without excision (*n* = 1) for progressive disease. The first patient in this group had a subtotal excision of atypical lipoma arising from the thigh, with postoperative scans revealing slow growth of residual tumor. The patient opted for reresection, 58.1 months after initial resection. This was followed by adjuvant radiation to 61.2 Gy in 34 fractions. The other patient underwent initial subtotal resection of an anterior compartment tumor of the leg, and surgery was not recommended due to potential functional morbidity (foot drop). He ultimately received radiation to 70 Gy in 2 Gy fractions to residual tumor, measuring 18.2 × 6.5 × 3.4 cm in size. This was delivered with a combination of photons and protons, 46.3 months after initial resection, due to slow increase in size. Posttreatment scans showed no further tumor growth.

Three patients had surgery with extent of initial resection unknown. One patient received excision, chemotherapy, and reexcision (all delivered in Greece; details of surgical extent and chemotherapy are unknown), followed by local recurrence after 27.4 months, which was treated with preoperative RT and excision. The second patient underwent two excisions in a span of 12 years, followed by local recurrence 10.3 years after last resection, also treated with preoperative RT and excision. The third patient underwent three excisions within a span of five years, followed by local recurrence 5 years after last resection, and was managed with excision and brachytherapy with RT delivered five days postoperatively with low-dose rate Ir-192 brachytherapy to a dose of 60 Gy prescribed to 5 mm from the plane of the implant.

## 3. Results

Median followup from time of last radiation treatment was 65.1 months (range, 3.7–196.0 months; average 73.1 months). Local tumor control was achieved in all 13 patients treated with radiation. There were no cases of metastatic disease or tumor dedifferentiation on followup. Summary of treatment is shown in Table 2. 

One patient, who received preoperative RT (50 Gy in 2 Gy fractions), developed a postoperative wound infection, requiring hospitalization and administration of IV antibiotics two months after surgery. She recovered fully. 

No cases of grade 3 or higher late toxicity from radiation were observed. There were two patients who developed grade 1 lower extremity edema in the treated limb. This includes the patient with grade 3 infection, as detailed above, and a patient who received resection and postoperative RT after initial gross total resection. One patient developed grade 1 telangiectasias; this patient had received brachytherapy to 60 Gy. Another patient, who was treated at initial presentation with subtotal excision followed by adjuvant RT (60 Gy in 2 Gy fractions), developed grade 2 sciatic neuropathy, requiring narcotics for pain control. Of note, she had tumor overlying the sciatic nerve at presentation. 

## 4. Discussion

Atypical lipomas are well-differentiated, slow-growing tumors that typically occur in the trunk and extremities and have a long natural history. Although surgery is the primary treatment modality, the likelihood of local recurrence is considered to be significant. It is difficult to estimate the true rates of local failure from published series due to the small number of patients and heterogeneity in tumor classification, but reported rates range from 8–52% (Table 3) [1, 3, 5–10, 12, 14, 15]. Rates of tumor dedifferentiation range from 0 to 13% in published reports [3–6, 8–10, 13–15]. No cases of tumor dedifferentiation occurred in this present series. 

Subclassification of atypical lipomas has been explored by several series in an effort to identify factors associated with local recurrence; however, due to small sample sizes, results have been inconclusive. One report by Kooby et al. [3] reviewed 91 lipomatous tumors and identified sclerosing histology to be associated with higher likelihood of local failure. A positive margin was also identified to be associated with increased failures, suggesting these subsets of patients should receive reexcision and consideration of adjuvant treatment. 

Standard management of local recurrence is reexcision. However, RT may have a role in providing long-term local control, particularly in areas where wide margins are challenging to achieve. To our knowledge, to date, there has not been any literature focusing on the use of RT in management of recurrent or incompletely resected atypical lipoma.

In this paper, eleven patients were treated for recurrent disease. All patients initially received surgical resection as primary treatment, with average time to recurrence of 63.2 months. Of this group, ten received a combination of RT and surgery and one received RT alone. At median followup of 48.7 months, all patients remained free of disease, demonstrating that long-term control can be achieved with the addition of RT.

 Two patients received adjuvant RT at initial presentation due to gross residual disease. At last followup (of 196.0 and 81.0 months, resp.), the patients remained free of recurrent disease, suggesting that long-term local control can be achieved with RT even in the setting of residual tumor.

The potential long-term morbidity of RT and surgery is important to consider when devising a management strategy. One patient, who received 60 Gy of adjuvant radiation following subtotal resection, developed late grade 2 sciatic neuropathy. Preoperative RT is typically delivered to a lower dose, typically 50 Gy, and carries less risk of late fibrosis [16] with long-term control equivalent to postoperative RT. Of patients with recurrent atypical lipomas, 6 received preoperative RT followed by gross tumor resection, and 3 underwent surgery followed by adjuvant RT. No grade 3 or higher late toxicity from radiation was observed, and all patients had control of their tumors.

In conclusion, atypical lipomas are slow-growing tumors with a propensity to recur locally; thus, long-term followup is recommended. RT can provide durable local control for patient and is recommended for patients at especially high risk of recurrence, due to multiply recurrent disease or residual gross tumor. For recurrent tumors, our results suggest a combination of reexcision and RT can provide long-term local control with acceptable morbidity. In the setting of gross residual disease, adjuvant RT can be considered to prevent local progression.

## Figures and Tables

**Table 1:** Individual patient and treatment details.

Sex	Age at diagnosis (Y)	Site	Initial management	Size of initial lesion	Time to LR/treatment of PD (M)	Treatment for progression/LR	Size of recurrent lesion	Total RT Dose (Gy)/fractions	Modality	FU (M)	Toxicity
F	47	Thigh	GTR	6.0 × 6.0 × 6.0	20	Preop RT + GTR	5.0 × 3.1 × 0.5, 2.0 × 1.0 × 0.5	50/25	Ph	35.4	
F	76	Thigh	GTR	4.0 × 3.0 × 1.5	20.2	Preop RT + GTR	10.0 × 8.7 × 2.7	50/25	Ph	47.2	Post-op wound infection requiring IV abx, Grade 1 edema
M	65	Forearm	GTR	15.0 × 5.0 × 5.0	36.7	Preop RT +GTR	8 × 7 × 3	50/25	Ph	7.5	
F	73	Thigh	GTR	6.0 × 9.0	124.2	GTR + postop RT	12.5 × 8.3 × 8.0	66/33	Ph	79.8	Grade 1 edema
F	45	Thigh	GTR	10.5 × 6.5 × 2	133.8	GTR + postop RT	14.0 × 8.0 × 4.1	50/25	Ph	147.2	
F	39	Thigh	Resection, followed by LR at 4 y, managed with GTR	Unknown	16.3	Preop RT + GTR	7.0 × 6.0 × 35	50/25	Ph	68.1	
M	46	Thigh	Resection, followed by LR at 11 y, treated with re-excision	Unknown	124.5	Preop RT + GTR	19 × 10.5 × 4.0	50/25	Ph	3.7	
F	55	Forearm	Resection, followed by LR at 2 y, treated with re-excision; followed by LR at 3 y, treated with re-excision	Unknown	87.9	GTR + brachytherapy	8.5 × 4.5 × 1.4	60/1	Ir-192	148.9	Grade 1 telangiectasia
M	41	Deltoid	STR + chemotherapy + re-excision	Unknown	27.4	Preop RT + GTR	11 × 9 × 14.5	50/25	Ph	21.3	
F	58	Thigh	STR	35.0 × 14.0 × 7.0	58.1	GTR + postop RT	Unknown	61.2/34	Ph	65.1	
M	66	Thigh	STR	20.0 × 18.0 × 3.0	46.3	RT alone	18.2 × 6.5 × 3.4	70/35	Ph + Pr	48.7	
F	36	Thigh	STR + postop RT	21.0 × 16.0 × 2.8	N/A	N/A	N/A	60/30	Ph	196.0	Grade 2 sciatic neuropathy

F indicates female, M: male; Y: years; GTR: gross total resection; STR: subtotal resection; RT: radiation; N/A: not applicable; LR: local recurrence; PD: progressive disease; M: months; Gy: Gray; Ph: photons; Pr: protons.

**Table 2:** Treatment and follow-up summary.

Sex	Age at diagnosis (Y)	Site	Initial management	Size of initial lesion	Time to LR/treatment of PD (M)	Treatment for progression/LR
F	47	Thigh	GTR	6.0 × 6.0 × 6.0	20	Reop RT + GTR
F	76	Thigh	GTR	4.0 × 3.0 × 1.5	20.2	Reop RT + GTR
M	65	Forearm	GTR	15.0 × 5.0 × 5.0	36.7	Reop RT + GTR
F	73	Thigh	GTR	6.0 × 9.0	124.2	GTR + postop RT
F	45	Thigh	GTR	10.5 × 6.5 × 2	133.8	GTR + postop RT

RT indicates radiation; GTR: gross total resection, STR: subtotal resection, FU: follow-up, M: months.

**Table 3:** Summary of outcomes reported for atypical lipomas in select series.

Author	Year	No. of patients	Mean FU (M)	LR	Dedifferentiation
				*n* (%)	*n* (%)
Evans et al. [12]	1979	22		9 (41%)	0
Azumi et al. [7]	1987	48	84	7 (15)	0
Weiss and Rao [8]	1992	46	108	20 (46)	3 (7)
Lucas et al. [9]	1994	32	112	15 (47)	6 (19)
Rozental et al. [13]	2002	31	84	16 (52)	2 (6)
Kooby et al. [3]	2004	91	47	20 (22)	3 (3)
Bassett et al. [14]	2005	51	52	14 (27)	1 (2)
Sommerville et al. [15]	2005	61	50	5 (8)	0
Evans [5]	2007	11^a^	>120	1 (9)	0
Serpell and Chen [10]	2007	6^b^	18	3 (50)	1 (17)
Billing et al. [4]	2008	38	90	4 (10)	0
Mavrogenis et al. [6]	2011	67	81	5 (11)	1 (2)

FU indicates follow-up, M: months, LR: local recurrence.

^a^Extremity lipomas only, ^b^atypical lipomas only.
